# The Manufacture and Characterization of Silver Diammine Fluoride and Silver Salt Crosslinked Nanocrystalline Cellulose Films as Novel Antibacterial Materials

**DOI:** 10.3390/gels7030104

**Published:** 2021-07-27

**Authors:** John Jackson, Claudia Dietrich, Ali Shademani, Adriana Manso

**Affiliations:** 1 Faculty of Pharmaceutical Sciences, University of British Columbia, Vancouver, BC V6T 1Z3, Canada; 2 Department of Oral Health Sciences, Faculty of Dentistry, The University of British Columbia, Vancouver, BC V6T 1Z3, Canada; claudiahelena.dietrich@ubc.ca (C.D.); amanso@dentistry.ubc.ca (A.M.); 3 Department of Microbiology and Immunology, The University of British Columbia, Vancouver, BC V6T 1Z3, Canada; 4 Department of Biomedical Engineering, University of British Columbia, Vancouver, BC V6T 1Z3, Canada; ali.shademani@mech.ubc.ca

**Keywords:** nanocrystalline cellulose, silver diammine fluoride, hydrogels, antibacterial, silver, poly vinyl alcohol

## Abstract

There is an unmet need for biocompatible, anti-infective, and mechanically strong hydrogels. This study investigated the use of poly vinyl alcohol (PVA), polysaccharides, and nanocrystalline cellulose (CNC) to deliver silver in a controlled manner for possible use against oral or wound bacteria. Silver was included in solvent cast films as silver diammine fluoride (SDF) or as nitrate, sulphate, or acetate salts. Hydrogel formation was assessed by swelling determinations and silver release was measured using inductively coupled plasma methods. Antibacterial studies were performed using Gram-positive and negative bacteria turbidity assays. PVA formed homogenous, strong films with SDF and swelled gently (99% hydrolyzed) or vigorously with dissolution (88% hydrolyzed) and released silver slowly or quickly, respectively. CNC-SDF films swelled over a week and formed robust hydrogels whereas CNC alone (no silver) disintegrated after two days. SDF loaded CNC films released silver slowly over 9 days whereas films crosslinked with silver salts were less robust and swelled and released silver more quickly. All silver loaded films showed good antibacterial activity. CNC may be crosslinked with silver in the form of SDF (or any soluble silver salt) to form a robust hydrogel suitable for dental use such as for exposed periodontal debridement areas.

## 1. Introduction

Infections associated with dental procedures remain a challenging problem for clinicians. Oral bacterial infections are particularly difficult to treat because there is a constant exposure to bacteria that is difficult to prevent [1]. Furthermore, systemic antibiotics may have limited access to infected areas such as in periodontal, periapical, or root canal systems. A global report, published in 2018, estimated the total worldwide cost due to dental diseases at US$ 544.41 billion [2].

Periodontal infections are often treated by gingival flap exposure, debridement, and surface treatment to encourage bone and gingival tissue regrowth [3], but bacterial infections in such procedures have frequent recurrence [4]. In dental implant sites (such as replacement screw-in teeth) the implant lies directly in the bone with a gingival flat covering, such that any initial infection, however small, may slowly grow, compromising prothesis and mucogingival health, which may require removal and a bone regrowth period before revision [5]. In root canal settings the drilled channel is usually filled with gutta percha and endodontic cement as a permanent material before crown fixation, yet this material has little or no antibiotic activity [6]. In dental caries lesion treatments, newer non-amalgam fillings often require revisions due to subsequent biofilm formation and recurrent caries lesions [7].

Silver has a strong antibiotic effect against both Gram-negative and Gram-positive bacteria [8] and a long history of use in dental settings. Silver and mercury amalgam materials have been successfully used for more than a century [9] with continuous prevention of secondary caries provided by the presence of silver on the material. In root canal settings, silver cones were used in the past as conical inserts in the root canal to effectively maintain a sterile environment instead of gutta percha [10]. However, the use of silver-containing dental amalgams is now avoided due to concerns over mercury toxicity [11] and the use in root canals stopped due to silver staining of tissues that could be visualized through gums. 

Silver diammine fluoride (SDF) has a long history of use as a solution applied to carious lesion sites, cavitated or non-cavitated, as an effective agent for minimal intervention with potential for reducing bacteria counting and arresting the caries progression. The material has been used in Brazil, Japan, and South America for many decades but has only recently been approved for use in Canada and the United States of America (USA) [12]. The increased awareness of the effectiveness of SDF in dental settings has seen the use of this material explode in clinics [13] and is used experimentally in noninvasive treatments and other sites. Although this material holds great promise as an anti-infective, its use is compromised by strong brown staining of demineralized and infected enamel and dentin. Dental hygienists will run floss through a solution of SDF before treating interproximal dental spaces of patients with gingival issues, and they must be careful not to run floss lines over the cheeks of patients as not to leave brown, tattoo-like staining on the skin. Although the stain on the skin disappears over time, the SDF manufacturers claim that the product does not stain healthy dental tissues.

There is insufficient research performed on controlled release systems for oral delivery of silver products. However, in two separate studies, Bromberg et al. [14] used poly(D,L-lactic-co-glycolic) acid (PLGA) wafers containing silver to treat periodontal disease and Balamuragan et al. [15] incorporated silver in dental bioglass for caries treatments.

Silver loaded biomaterials such as poly vinyl alcohol (PVA) [16,17], alginate [18], and carboxymethyl cellulose (CMC) [19] have been used extensively as controlled release anti-infective systems to treat wounds. These materials swell in aqueous settings to form hydrogels that are both comfortable medical devices for patients while providing a rapid but extended duration of silver release to prevent infection. Similarly, nanocrystalline cellulose (CNC) has been investigated as a silver delivery system for wound healing applications [18,19].

Clearly, there exists a need for robust anti-infective biomaterials in wound and dental settings. Considering the renewed interest in the use of silver products as anti-infective wound dressings and the use of SDF in dentistry, the objective of this study was to explore the use of swelling hydrogel—forming biomaterials such as hyaluronic acid (HA), CMC, PVA, and CNC as delivery systems for SDF in medical applications. 

## 2. Results

### 2.1. Film Morphology

Films made from SDF in CMC or HA failed to form homogenous materials. There was evidence of phase separation of solid silver from the polysaccharide material as witnessed by particulate dark colored debris left in clusters at the last point of drying. Further experiments with these polysaccharides were abandoned. 

SDF loaded films produced from PVA solutions (both the 99% and 88% hydrolyzed) were strong thin films (approx. 50 µm thick) with varying degrees of browning not seen in films without SDF. The addition of glycerol at 10% (*w*/*w* to PVA) allowed the films to be flexed without breaking. SDF loaded CNC films were opaque with a shiny black, brown sheen that appeared metallic (for both glycerol and non-glycerol films). These films could be rendered flexible by the addition of glycerol but would still snap under a strong load. 

**Figure 1:**
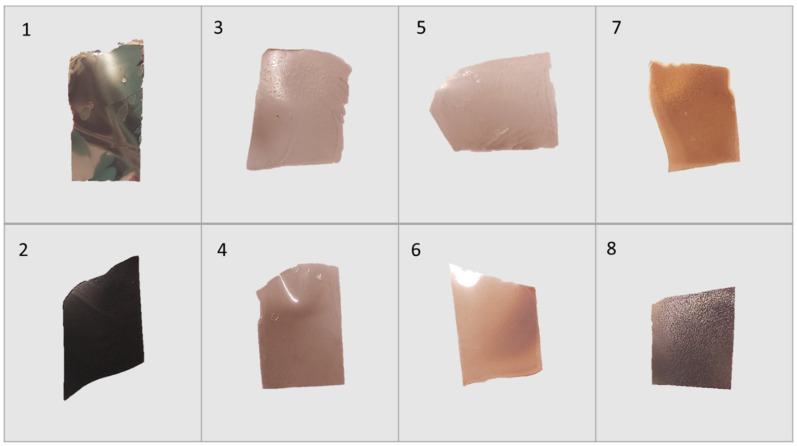
Appearance of CNC cast films including various silver salts. (**1**) SDF with CNC, (**2**) SDF with CNC and glycerol, (**3**) AgNO_3_ with CNC, (**4**) AgNO_3_ with CNC and glycerol, (**5**) Ag_2_SO_4_ with CNC, (**6**) Ag_2_SO_4_ with CNC and glycerol, (**7**) Ag Acetate with CNC, and (**8**) Ag Acetate with CNC and glycerol.

CNC films loaded with silver nitrate, sulphate, or acetate were only slightly discolored brown, and similar to SDF-CNC films, were made flexible by the addition of glycerol. Pictures of several of these films are shown in Figure 1.

### 2.2. Film Swelling

#### 2.2.1. SDF Films Swelling

**Figure 2:**
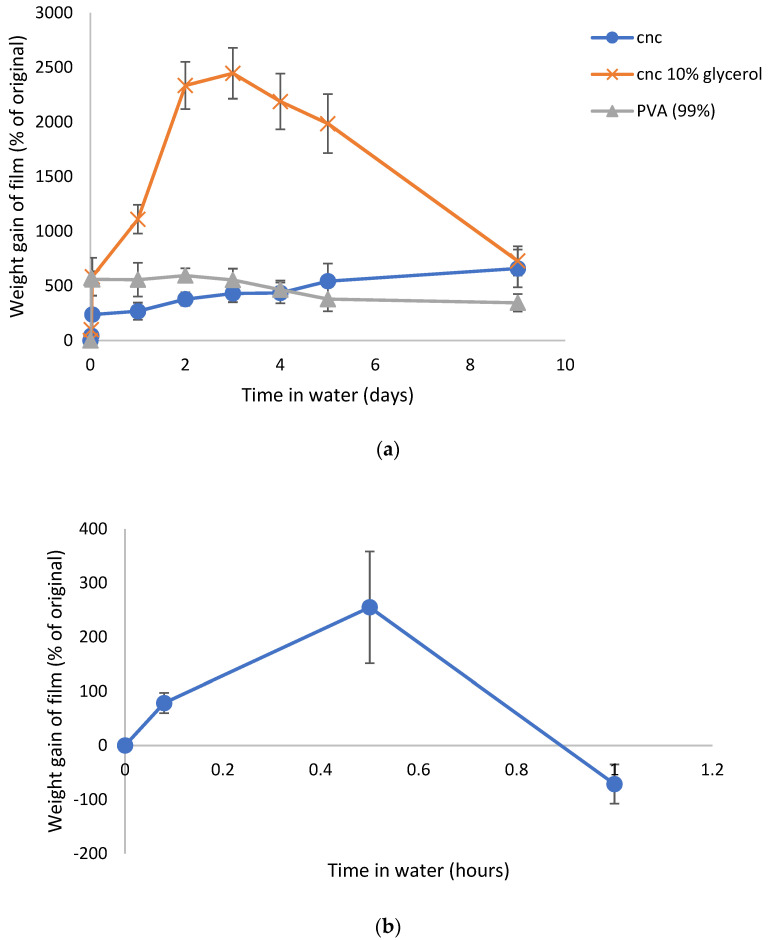
(**a**) Time course of swelling of SDF loaded CNC or PVA hydrogel films in water. (**b**) Time course of swelling of SDF loaded PVA (88% hydrolyzed) films in water.

PVA (99%) films swelled rapidly in water by 500% within several hours and then stabilized in that swollen state for many days with slight loss of weight after 9 days (Figure 2a). CNC films swelled much slower with an initial 200% increase followed by a slow almost linear swelling over the next 9 days to over 500%. These films had become firm hydrogels that could be physically moved on the filter as a single monolithic block with the consistency of wet pasta. The addition of glycerol to the CNC films allowed the films to swell continuously for over 3 days to almost 2500% of their original size. After that time, the films began to physically disintegrate, and small fragments of the materials were lost from the films on the Millipore filter. The swelling data for these films is shown in Figure 2a. SDF loaded PVA (88% hydrolyzed) swelled over 30 min but then dissolved over one hour, as shown in Figure 2b.

#### 2.2.2. Silver Salt-CNC Film Swelling

**Figure 3:**
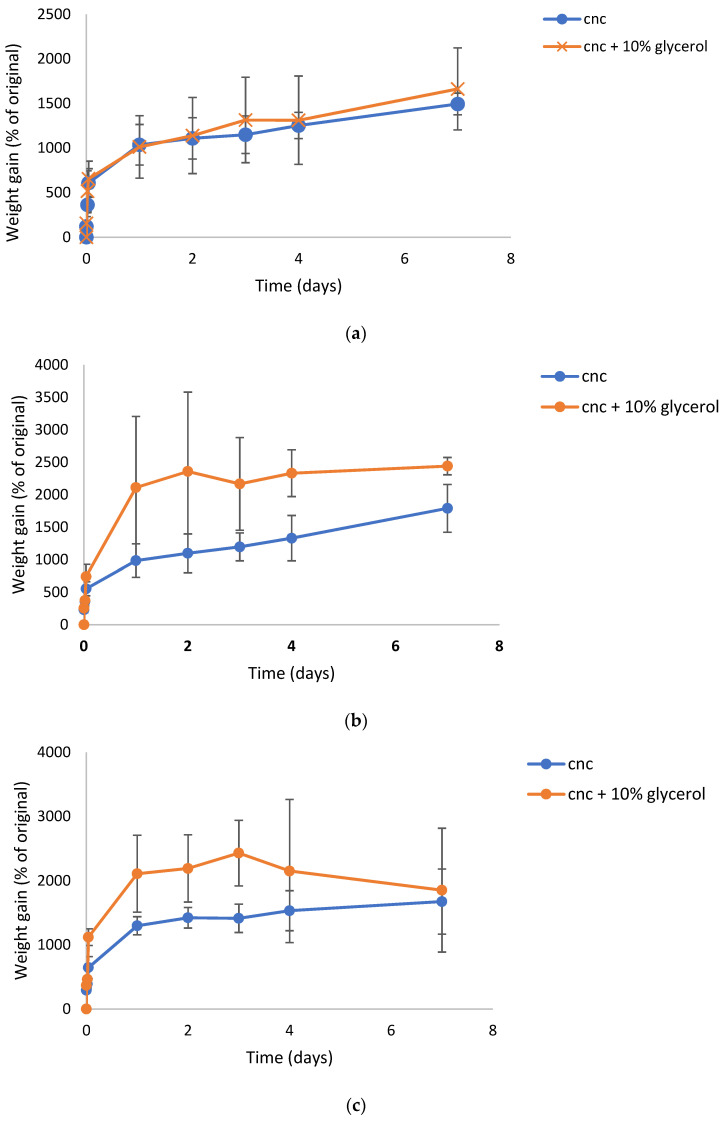
(**a**) Time course of swelling of silver nitrate loaded CNC hydrogel films in water. (**b**) Time course of swelling of silver sulphate loaded CNC hydrogel films in water. (**c**) Time course of swelling of silver acetate loaded CNC hydrogel films in water.

CNC films loaded with silver nitrate, silver sulphate, or silver acetate at 2% silver loadings (similar to SDF loadings in CNC) all swelled in a similar manner to the SDF films with an initial fast swelling of approximately 500% (higher than CNC-SDF) with a further slow increase in swelling over 7 days, as seen in Figure 3a–c. The addition of 10% glycerol increased the degree of swelling for CNC-SDF films. For all three silver salts, this glycerol containing hydrogel films lost integrity in less than two days and existed more as viscous solutions without evidence of gel crosslinking. For non-glycerol containing films, integrity was maintained for a longer period (possibly 1–2 days) but the films were never robust to the level of SDF crosslinked films and by day 7 they existed as viscous solutions.

#### 2.2.3. Effect of Silver Concentration on CNC Film Swelling and Hydrogel Integrity

**Figure 4:**
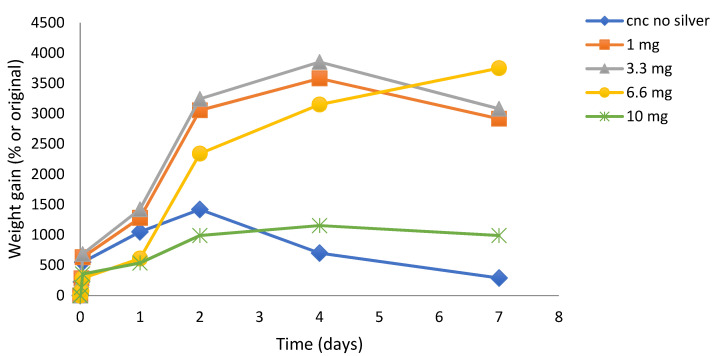
Time course of swelling of SDF loaded CNC hydrogel films (1–10 mg) in water. Loadings describe weight of silver in 500 mg manufactured films, thus 10 mg is equivalent to 2% *w*/*w* silver loading in CNC.

**Figure 5:**
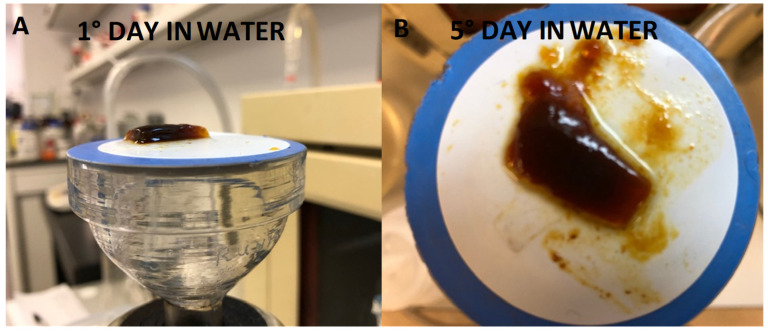
Swollen appearance of SDF loaded CNC film. (**A**) day 1; (**B**) day 5.

SDF: The crosslinking effect (slower, continuous, and reduced swelling with robust hydrogel) of SDF on CNC film formation was concentration dependent, as shown in Figure 4. Using 10 mg SDF loadings, the films were robust and swelled in a steady manner over one week (similar to data shown in Figure 2a). Using a 6.6 mg silver loading, the CNC films swelled more than the 10 mg, but after 2 days the films were less robust, looser, and gel-like. For lower silver loadings (3.3 and 1 mg), the films swelled massively over 3–4 days to over 3500% but started to physically disintegrate after that. Furthermore, these low silver-load films were more similar to viscous solutions after 1 day with no evidence of crosslinking. Pictures of swollen SDF (10 mg) crosslinked films at day 1 and day 5 are shown in Figure 5A,B.

**Figure 6:**
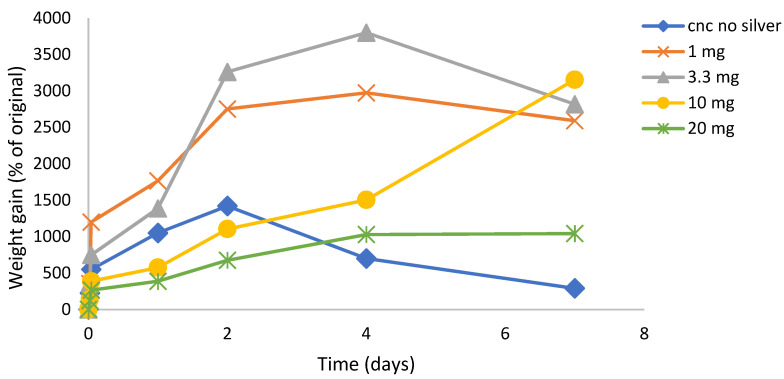
Time course of swelling of silver nitrate loaded CNC hydrogel films in water using various loadings of silver nitrate. Loadings describe weight of silver in 500 mg manufactured films, thus 10 mg is equivalent to 2% silver loading.

**Figure 7:**
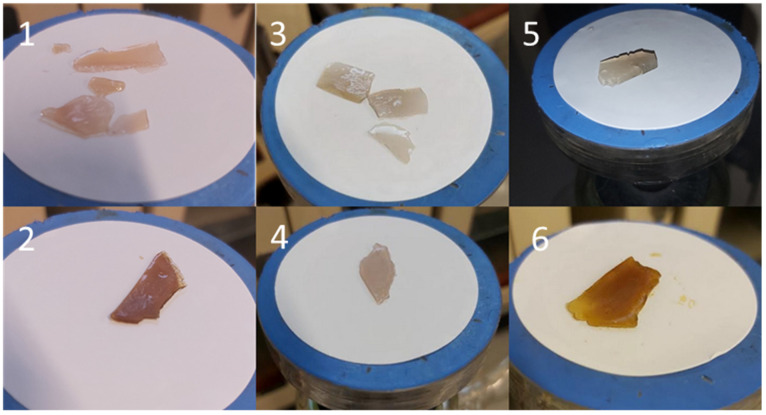
Swollen appearance of various silver salt loaded CNC films. (**1**) silver nitrate with CNC, (**2**) silver nitrate with CNC and glycerol, (**3**) silver sulphate with CNC, (**4**) silver sulphate with CNC and glycerol, (**5**) silver acetate with CNC, (**6**) silver acetate with CNC and glycerol.

Silver Nitrate: similar concentration dependent effects were observed for silver salt effects in CNC films but at higher silver loadings as seen in Figure 6. Thus, when using 20 mg of silver, the degree of swelling was similar to that measured for 10 mg of SDF silver (Figure 4) and stable between 4 and 7 days. At lower silver loadings, all films swelled greatly and for 3.3 and 1mg loadings, the films began to quickly disintegrate after several days, as seen by the apparent reduced swelling (weight loss). At the higher 20 mg loadings the films had more reduced integrity than SDF crosslinked films. However, in comparison to SDS-CNC films, there was little evidence of brown coloration in these films at the time of manufacture (Figure 1) and during the release study as swollen gels. Pictures of swollen silver salt films at day 7 are shown in Figure 7.

### 2.3. Silver Release Studies

#### 2.3.1. SDF—Or CNC Studies

**Figure 8:**
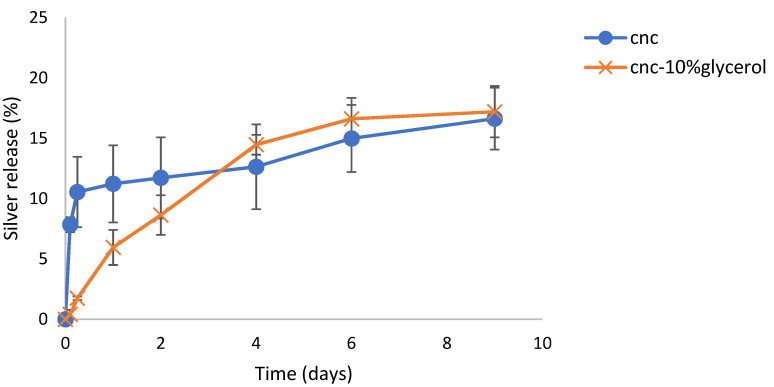
Time course of the release of silver from SDF loaded CNC hydrogels films in water.

The time course of silver release from SDF loaded CNC films is shown in Figure 8. CNC loaded films released silver in a sustained manner over 9 days. For CNC alone, the release was characterized by approx. 11% released in the first day followed by a steady almost linear release over the next 8 days. For CNC + glycerol, silver was released in a more continuous fashion without a burst phase of release so that by day 9 more than 15% of the loaded silver had been released.

#### 2.3.2. Silver Salt-CNC Studies

**Figure 9:**
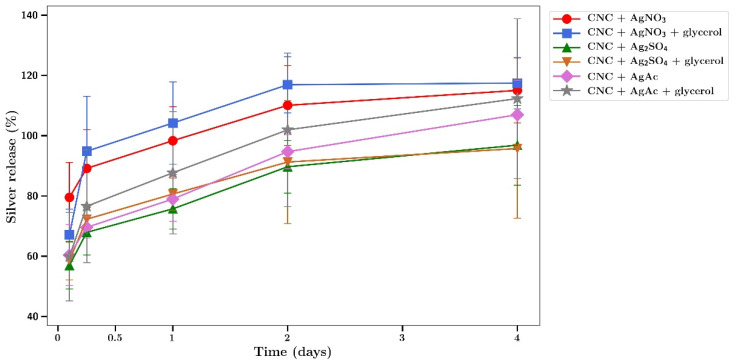
Time course of the release of silver from various silver salt loaded CNC hydrogels with or without glycerol in water.

Silver release from silver salt loaded CNC films was quicker than for SDF. The time course of silver release from silver salt loaded CNC films was characterized by a fast sustained release of silver with essentially all silver released by 2 to 4 days (Figure 9). There was no effect of adding glycerol on these release profiles.

### 2.4. Bacterial Studies

**Figure 10:**
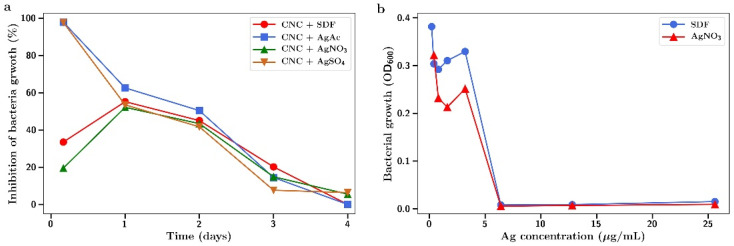
Inhibition of MRSA growth by (**a**) release media from incubation of SDF or silver salt crosslinked CNC at 4 h, 1, 2, 3, and 4 days; (**b**) MRSA growth as a function of silver concentration using SDF or silver nitrate in solution.

**Figure 11:**
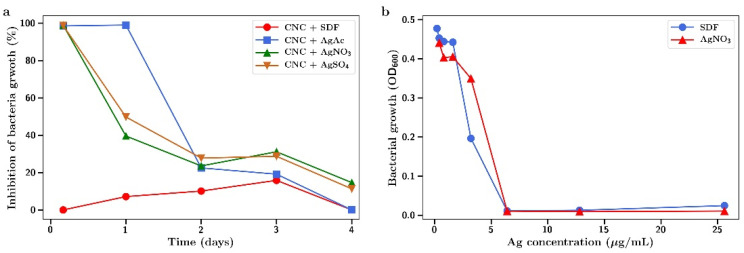
Inhibition of *E. coli* growth by (**a**) release media from incubation of SDF or silver salt crosslinked CNC at 4 h, 1, 2, 3, and 4 days; (**b**) *E. coli* growth as a function of silver concentration using SDF or silver nitrate in solution.

Both MRSA and *E. coli* were found to be highly susceptible to the antibacterial effects of SDF or silver salts as shown in Figure 10 and Figure 11. The salts and SDF were equivalent in potency with full inhibition of bacterial growth at 7 µg/mL for both bacteria (Figure 10b and Figure 11b). CNC films that had been crosslinked with SDF or silver nitrate, sulphate, or acetate and were then incubated in media which was collected (and replaced with fresh media) at 4 h, 1, 2, 3, and 4 days. This media was then included in bacterial incubations to investigate the antibiotic activity. 

For MRSA, the release media from the 4 h incubation fully inhibited bacterial growth for silver sulphate and acetate but not for SDF or silver nitrate release media (Figure 10a). In one day, all incubation media from silver salts was equivalent in producing approximately 50% inhibition which fell in an almost linear fashion over the next few days to no effect by day 4. 

For *E. coli*, the 4 h release media for all three silver salt incubations fully inhibited bacterial growth (Figure 11a). This was extended to day 1 for silver acetate incubations. After day 1, all silver salt release media showed a declining antibacterial effect dropping to between 0% and 15% by day 4. The release media from SDF incubations showed little antibacterial effects until day 3 (15% inhibition of *E. coli* growth).

## 3. Discussion

Silver is among the numerous metals that have antibacterial properties. However, unlike other metals, silver has low toxicity in humans [20], making it attractive for use in the clinic with an increasing prevalence of drug resistant bacteria. In particular, silver nanoparticles have shown to be promising in wound healing, implantations, wound disinfection, sensing, catalysis, the coating of other surfaces, etc. [21].

A silver product that has been recently approved for use in dentistry by the FDA is silver diammine fluoride (SDF). The simplicity and affordability of SDF treatment has gained attention within the past decade. Clinical trials show that SDF prevented and arrested coronal caries in primary teeth in preschool children and in root surface of permanent teeth in adult [22]. Recent systematic reviews of human clinical trials indicate that silver diamine fluoride may be a simpler anticariogenic agent than fluoride alone [23]. The mechanism of action of SDF is hypothesized to arise from its anticariogenic properties and its ability to extend enamel surface microhardness and reduce enamel surface mineral loss [24]. Neither the mechanisms of action nor their optimizations are well understood.

SDF has been used for many years in countries such as Brazil in dentistry to prevent caries progression, but the potential of the product and its application in other areas has not been fully explored. While it is currently of interest to inhibit caries, there are many situations in dentistry in which a biocompatible packing type material that can release silver ions in a controlled manner is useful. These areas may include root canal channels or periodontal pockets. Similarly, biocompatible materials that form hydrogels to release silver may be useful for wound dressing purposes. 

Although 12% and 38% *w*/*v* SDF solutions are available within the commercial market, most SDF products are prepared at a degree of 38%. Studies have revealed that 12% SDF isn’t as effective as 38% SDF in arresting caries among children [22,25]. A particular problem associated with the use of SDF or many silver salts such as silver nitrate on teeth is brown staining. An advantage of prebinding the silver agents to CNC may be that the staining occurs on the CNC prior to addition to a dental setting, thus protecting the dental surface. Although SDF is not currently used in wound healing compositions, this avoidance of tissue staining can be advantageous for use in that field too. It was previously reported that demineralized teeth stain darkly when SDF contacts the surface due to silver chloride and metallic silver production [25]. It is possible that the dark metallic staining observed when SDF binds to CNC is due to a similar process. This may be highly advantageous for using SDF as a controlled release system in dental or medical settings since other workers have shown that nanosilver fluoride, which is already colored, does not stain teeth but shows good inhibition of caries bacterial growth [26]. For this reason, the use of silver diamine fluoride in combination with biocompatible polymers was investigated as potential controlled release antibiotic systems. PVA is a synthetic, biodegradable, biocompatible, water-soluble polymer utilized in medical applications such as wound dressings, artificial skin, coatings, transdermal patches, cardiovascular devices, and drug delivery systems [27]. As the degree of hydrolysis of the PVA increases, such does the resistance to dissolution [28]. PVA was used in this study in either the 99% or 88% hydrolyzed forms. Previous studies have shown that silver salt loaded films made from 99% PVA swelled slightly but did not dissolve, whereas PVA films made from 88% PVA swelled and dissolved quickly [29]. In this study, similar results were observed using SDF in PVA films, whereby the 88% hydrolyzed films swelled over the first 30 min but then quickly dissolved afterward (Figure 2b). However, the SDF loaded 99% hydrolyzed films rapidly swelled by approximately 600% and maintained that degree of swelling for a few days before slowly losing weight over the next week. This type of swelling is consistent with previous reports [29] and may simply reflect the low solubility of the 99% version of the PVA. The PVA films turned brown to a certain degree as this indicates silver nanoparticle formation in situ, as previously described [28,29]. A more intense browning occurred with CNC-SDF films using the same SDF loading such that this nanoparticle formation effect was probably minor in PVA. 

Nanocrystalline cellulose is a material recently extracted from microcrystalline cellulose and represents the shortest polymer chains of cellulose. As such, the material has a high surface area to volume ratio and the surface is covered with free hydroxyl groups. Similar to carbon fiber, the material has immense strength when incorporated into composites. This material has been scrutinized for numerous physical and biomedical applications [30,31,32]. In this laboratory, CNC was previously shown to bind and release drugs in a pharmaceutically relevant manner supporting the idea of the use in CNC in medical applications, especially considering the established biocompatibility of cellulose [16]. In these studies, suspensions of CNC with no SDF dried to a thin translucent film that could be gently handled with forceps, but of which were brittle. These films alone showed signs of interchain bonding because, when rehydrated, the fibers did not immediately or freely resuspend but instead, behaved like a partially crosslinked material, whereby the film swelled greatly (1400%, Figure 6) over the first two days, followed by a steady break, and then up to 250% swollen at day 7. The situation was dramatically changed by the inclusion of SDF (10 mg), whereby the dried films had a dark metallic opaque sheen and were robust when handled. In water, these films swelled slowly (Figure 2a) and continuously from approximately 250% to 600% over 7 days and the hydrogels remained strong and handleable. Other workers have described similar effects of non-silver metal ion interactions with underivatized CNC, whereby the strength of the swollen material increased and the degree of swelling decreased [33,34,35]. Similarly, Hossein et al. [36] showed that surface derivatized crosslinked CNC swelled less in water than un-crosslinked CNC. The concentration dependent nature of this crosslinking was confirmed using lower amounts of silver, which were associated with more swelling and subsequent break up (1 and 3.3 mg SDF, Figure 4). These properties clearly demonstrate a strong crosslinking effect of SDF on the CNC chains similar to those previously reported by our group for silver on PVA 88% with heat [28]. The robust swollen nature of the SDF-CNC films at day 1 and 5 are shown in Figure 5. The inclusion of 10% glycerol in polysaccharide films has been shown to increase flexibility [17] and this was found to be true for CNC in this study. The inclusion of glycerol resulted in a large increase in the degree of swelling of the CNC films over the first 2 days (2400%, Figure 2a) which was then reduced to the same level as the non-glycerol SDF-CNC films of approximately 600% at day 7. It is likely that the glycerol molecules disrupted the SDF-CNC crosslinking effect to some degree and allowed the increased penetration of water into the films. These levels of swelling may be acceptable for surface wound healing dressings (such as diabetic ulcer treatment) but are excessive for dental applications such as implant or root canal cavities or periodontal films. It is likely that for such dental applications, the CNC-SDF films can be used without glycerol as a certain level of firmness is needed for accurate placement. 

CNC films cast using silver ions from silver nitrate, sulphate, or acetate in solution had only mild browning effects on drying. These browning effects were insignificant in comparison to those caused by SDF. Silver nitrate loaded dried PVA films were transparent and only slightly brown but heating resulted in increased browning, as well as crosslinking for PVA 88% [28]. Whereas pure CNC dried films swelled (2 days) but broke up over 7 days, indicating only mild molecular interactions. Silver salt containing films swelled in a similar manner to approximately 1000% on day 1 and rising to 1500% on day 7 with no signs of breakdown at that time (Figure 3a–c). The addition of glycerol had little effect on these swelling data. These data clearly indicate a strong crosslinking effect of silver on CNC and the lack of strong browning indicates only a minor level of silver nanoparticle formation. 

The strength of the silver nitrate crosslinking was found to depend on the silver concentration, as shown in Figure 6. Low concentrations of 1 and 3.3 mg produced CNC films that swelled quickly to approximately 1500% by day 1, rose to approx. 3000 by day 4, and then began to decline, indicating the start of film breakdown due to weak crosslinking. However, using 10 or 20 mg caused slow swelling that continued to increase for 10 mg concentrations but plateaued at approximately 500% for 20 mg silver nitrate, indicating a strong stable crosslinking effect. 

When comparing SDF and silver salt crosslinking effects, the studies performed using various concentrations of SDF in the 1–10 mg range showed similar effects to salts, except at approximately half the silver concentration (Figure 4). Accordingly, films crosslinked with silver at 10 mg (as compared to 20 mg for silver nitrate) plateaued and remained swollen at approximately 1000%. 

These degrees of swelling of CNC seen with silver salts are greater than those observed for SDF-CNC films (Figure 2a, 250% day 1 to 500% day 7) and suggest weaker intermolecular forces as compared with SDF effects. This is supported by the observation that the addition of glycerol to SDF-CNC films allowed for greater swelling above 2000%, whereby the glycerol molecules may sterically hinder any close association between cellulose and SFD molecules. 

The antibacterial effects of silver as either a salt or in the SDF form were confirmed against two difficult-to-treat bacteria; Gram positive drug resistant MRSA and Gram negative *E. coli* (Figure 10 and Figure 11). The antibacterial effects observed at concentrations in the 1–7 µg/mL range are similar to those reported elsewhere [37,38]. Using functionally crosslinked CNC loaded with silver nanoparticles, other workers have shown these hydrogels to be antibacterial against Gram negative [39,40] and Gram positive [39] bacteria. 

The release media from both silver salt and SDF-CNC hydrogel incubations were also found to have various degrees of antibacterial effects (Figure 10 and Figure 11). The lower inhibitory effects of SDF match the reduced silver release profile for SDF from CNC, as shown in Figure 8. These studies are comparative and qualitative between salts and SDF where the hydrogels are incubated in a large volume of water, thereby significantly diluting the silver concentration. In dental or wound settings, the volume of locally available aqueous media is greatly reduced so that silver concentrations are higher than those found here. 

In conclusion, these data clearly illustrate the potential of silver crosslinked nanocrystalline cellulose as controlled release formulations of silver for extended antibacterial effects in dental or wound settings. The lower level of swelling, the robust nature of the hydrogel, and the slow release profiles of silver from SDF crosslinked CNC make such compositions excellent candidates for use in medical settings requiring extended antibiotic treatment. In particular, these silver loaded, robust hydrogels that cover wounds may be comfortable for patients and may offer a much improved anti-infective system over the existing silver loaded dressings that are physically removed, often with painful and wound damaging consequences. Alternatively, less swollen SDF-CNC gels may form novel anti-infective support or filling structures in dental settings.

## 4. Materials and Methods

Carboxymethyl-cellulose high molecular weight was obtained from Fluka (Ottawa, ON, Canada). PVA (99% hydrolyzed mol wt 125 KDa. and 88% hydrolyzed 150 KDa known as Selvol 540 and Selvol 125) was obtained from Sekisui (Jersey City, NJ, USA). Hyaluronic acid high molecular weight was obtained from Lifecore (Chaska, MN, USA). Silver Diammine Fluoride, Advantage Arrest^®^, was obtained from Elevate Oral Care (West Palm Beach, FL, USA). and contained 25% by weight of silver. Silver nitrate, sulphate and acetate were obtained from Sigma chemicals (St. Louis, MO, USA). CNC was obtained from Alberta Innovates (Edmonton, Alberta, Canada). In all experiments, silver is described as elemental silver so that for example 10 mg of silver equates to a weight of 15.8 mg of silver nitrate.

### 4.1. Film Casting

Films were cast as previously described [28,29]. Briefly, solutions of PVA were made up at 10% (*w*/*w*) in water by boiling and stirring for 1 h or until all the PVA was dissolved in the solution and allowed to cool. Other solutions were produced at 2.5% (*w*/*w*) by stirring at room temperature. Nanocrystalline cellulose was used as provided as a colloidal suspension at 2% (*w*/*w*). Twenty mL of either HA, PVA, CMC, or CNC solutions or suspensions were pipetted into 20 mL glass vials and SDF was included by pipette, such that 10mg of silver was included at 2% (*w*/*w*) to polymer or CNC. In certain vials, 10% glycerol was added (by weight to PVA). In other experiments silver was added at a final weight of 2% (ionic silver to polymer or CNC). All vials were capped and tumbled to ensure all contents were mixed or dissolved. The entire 20 mL was then poured into 5 cm diameter plastic petri dishes and placed in an oven at 37 °C with the petri lid partially covering the contents to prevent rapid evaporation. After 24 h, the films were removed from the petri dish by gentle teasing with forceps. 

Several experiments investigated the relationship between the amount of silver added and the degree of CNC swelling. In these experiments with SDF, either 1, 3.3, 6 or 10 mg of silver was added to the CNC suspension to give 0.2%, 0.66%, 1.2%, or 2% final silver concentrations in the dry CNC films, respectively. For the same experiments using silver nitrate, similar silver concentrations were used but with up to 20 mg silver (4% silver to CNC).

### 4.2. Swelling Experiments

Swelling experiments were performed as previously described [17,28,29]. Briefly, 25 mg sections of film were weighed and placed on preweighed moist Millipore filter discs. The combination was placed on a sintered glass filter head connected to a 1-L flask. The filter and film were then moistened with excess water and after 2 min the vacuum was applied for 10 s (enough to pull excess water from the set up without drying the Millipore filter). The moist film and filter combination were then reweighed, recorded, and placed in a petri dish with a small amount of water enough to keep the film wet. At appropriate times, the filter and film were removed from the device and the vacuum procedure was repeated with weighing. The swelling of the film was then calculated as the % increase in weight of the film over time.

### 4.3. Silver Release Experiments

Silver release studies were performed as previously described [28]. Briefly, 25 mg sections of films were placed in 15 mL glass tubes with 5 mL of water and placed in a sealed 37 °C incubator. At different time points (baseline, 30 min, 1 h, 5 h, 1 d, 2 d, 3 d, 4 d, 1 w, 2 w, 3 w), all the water was removed and saved. A fresh 5 mL aliquot of water was carefully placed into the tube on top of the hydrogel film and the tubes were placed back into the incubator. All samples were then stored at 4 °C until analysis. Silver released into the water was quantitated using an inductively coupled plasma analyzer (ICP) (Agilent 725 ICP-OES USA) using silver nitrate calibration standards in the range 10 to 0.005 µg/mL.

### 4.4. Observational Methods

Films were subject to optical examination using stereo and high magnification microscopy (Olympus microscopes) and the coloration and presence of precipitated materials in the films were observed. Photographs of films in swollen states were taken at appropriate time points for optical visualization of the hydrogel film. The integrity of swollen hydrogel films was assessed using a tiny spatula to observe if the film remained intact as it was moved on the Millipore filter. 

### 4.5. Bacterial Methods

#### 4.5.1. Bacterial Inoculation

*Escherichia coli* clinical isolate K12 (ATCC^®^ 25922) and methicillin resistant *staph. aureus* (MRSA) USA300 (ATCC^®^ BAA-1556™) was purchased from American Type Culture Collection (ATCC, Manassas, VA, USA) and used for the bacterial experiments. The bacteria were grown at 37 °C in Luria-Bertoni (LB) broth (Difco, Sparks, MD, USA) overnight in an orbital shaker, and the optical density (OD) was measured at 600 nm.

#### 4.5.2. In Vitro Study of the Released Media and Silver

A subculture of each strain at OD_600_ of 0.0025 was prepared of which 100 µL was added to 100 µL released media samples from day 1 to 4 in a 96-well plate assay. Furthermore, the inhibitory activity of silver released from SDF and AgNO_3_ was investigated by aliquoting 100 µL of serial dilutions into 100 µL of subcultures of each strain. All assays were incubated for 24 h at 37 °C and analyzed using a Varioskan microplate reader (Thermo Scientific^TM^). Bacterial growth inhibition was evaluated with respect to the control wells’ growth for days 1–4 release samples. Silver assays were presented as the measured turbidity (OD_600_) of the wells.

## Data Availability

Not applicable.

## References

[1] Marcenes W., Kassebaum N.J., Bernabé E., Flaxman A., Naghavi M., Lopez A., Murray C.J.L. (2013). Global Burden of Oral Conditions in 1990–2010. J. Dent. Res..

[2] Righolt A.J., Jevdjevic M., Marcenes W., Listl S. (2018). Global-, regional-, and country-level economic impacts of dental diseases in 2015. J. Dent. Res..

[3] Mombelli A. (2018). Microbial colonization of the periodontal pocket and its significance for periodontal therapy. Periodontol. 2000.

[4] Cionca N., Giannopoulou C., Ugolotti G., Mombelli A. (2010). Microbiologic Testing and Outcomes of Full-Mouth Scaling and Root Planing With or Without Amoxicillin/Metronidazole in Chronic Periodontitis. J. Periodontol..

[5] Coli P., Christiaens V., Sennerby L., Bruyn H. (2017). De Reliability of periodontal diagnostic tools for monitoring peri-implant health and disease. Periodontol. 2000.

[6] ElReash A.A., Hamama H., Eldars W., Lingwei G., Zaen El-Din A.M., Xiaoli X. (2019). Antimicrobial activity and pH measurement of calcium silicate cements versus new bioactive resin composite restorative material. BMC Oral Health.

[7] Jokstad A. (2016). Secondary caries and microleakage. Dent. Mater..

[8] Sharma G. (2015). Approaches to Arresting Dental Caries: An Update. J. Clin. Diagn. Res..

[9] Himmelberger L.K. (2015). Justifiable criticism and dental amalgam. J. Am. Dent. Assoc..

[10] Timpawat S., Jensen J., Feigal R.J., Messer H.H. (1983). An in vitro study of the comparative effectiveness of obturating curved root canals with gutta-percha cones, silver cones, and stainless steel files. Oral Surg. Oral Med. Oral Pathol..

[11] Hanson M., Plevab J. (1991). The Dental Amalgam Issue. A Review.

[12] Horst J.A. (2018). Silver Fluoride as a Treatment for Dental Caries. Adv. Dent. Res..

[13] Duangthip D., Jiang M., Chu C.H., Lo E.C.M. (2015). Non-surgical treatment of dentin caries in preschool children—Systematic review. BMC Oral Health.

[14] Bromberg L.E., Braman V.M., Rothstein D.M., Spacciapoli P., O’Connor S.M., Nelson E.J., Buxton D.K., Tonetti M.S., Friden P.M. (2000). Sustained release of silver from periodontal wafers for treatment of periodontitis. J. Control. Release.

[15] Balamurugan A., Balossier G., Laurent-Maquin D., Pina S., Rebelo A.H.S., Faure J., Ferreira J.M.F. (2008). An in vitro biological and anti-bacterial study on a sol–gel derived silver-incorporated bioglass system. Dent. Mater..

[16] Jackson J.K., Letchford K., Wasserman B.Z., Ye L., Hamad W.Y., Burt H.M. (2011). The use of nanocrystalline cellulose for the binding and controlled release of drugs. Int. J. Nanomed..

[17] Jackson J.K., Skinner K.C., Burgess L., Sun T., Hunter W.L., Burt H.M. (2002). Paclitaxel-loaded crosslinked hyaluronic acid films for the prevention of postsurgical adhesions. Pharm. Res..

[18] Bahadoran M., Shamloo A., Nokoorani Y.D. (2020). Development of a polyvinyl alcohol/sodium alginate hydrogel-based scaffold incorporating bFGF-encapsulated microspheres for accelerated wound healing. Sci. Rep..

[19] deBoer T.R., Chakraborty I., Mascharak P.K. (2015). Design and construction of a silver(I)-loaded cellulose-based wound dressing: Trackable and sustained release of silver for controlled therapeutic delivery to wound sites. J. Mater. Sci. Mater. Med..

[20] Hadrup N., Sharma A.K., Loeschner K. (2018). Toxicity of silver ions, metallic silver, and silver nanoparticle materials after in vivo dermal and mucosal surface exposure: A review. Regul. Toxicol. Pharmacol..

[21] Söderstjerna E., Bauer P., Cedervall T., Abdshill H., Johansson F., Johansson U.E. (2014). Silver and Gold Nanoparticles Exposure to In Vitro Cultured Retina—Studies on Nanoparticle Internalization, Apoptosis, Oxidative Stress, Glial- and Microglial Activity. PLoS ONE.

[22] Fung M.H.T., Duangthip D., Wong M.C.M., Lo E.C.M., Chu C.H. (2018). Randomized Clinical Trial of 12% and 38% Silver Diamine Fluoride Treatment. J. Dent. Res..

[23] Contreras V., Toro M.J., Eliás-Boneta A.R., Encarnación-Burgos A. (2017). Effectiveness of silver diamine fluoride in caries prevention and arrest: A systematic literature review. Gen. Dent..

[24] Buchalla W., Imfeld T., Attin T., Swain M.V., Schmidlin P.R. (2008). Relationship between Nanohardness and Mineral Content of Artificial Carious Enamel Lesions. Caries Res..

[25] Zhao I.S., Gao S.S., Hiraishi N., Burrow M.F., Duangthip D., Mei M.L., Lo E.C.-M., Chu C.-H. (2018). Mechanisms of silver diamine fluoride on arresting caries: A literature review. Int. Dent. J..

[26] Targino A.G.R., Flores M.A.P., dos Santos Junior V.E., de Godoy Bené Bezerra F., de Luna Freire H., Galembeck A., Rosenblatt A. (2014). An innovative approach to treating dental decay in children. A new anti-caries agent. J. Mater. Sci. Mater. Med..

[27] Galya T., Sedlařík V., Kuřitka I., Novotný R., Sedlaříková J., Sáha P. (2008). Antibacterial poly(vinyl alcohol) film containing silver nanoparticles: Preparation and characterization. J. Appl. Polym. Sci..

[28] Jackson J., Plackett D., Hsu E., Lange D., Evans R., Burt H. (2021). The Development of Solvent Cast Films or Electrospun Nanofiber Membranes Made from Blended Poly Vinyl Alcohol Materials with Different Degrees of Hydrolyzation for Optimal Hydrogel Dissolution and Sustained Release of Anti-Infective Silver Salts. Nanomaterials.

[29] Jackson J., Burt H., Lange D., Whang I., Evans R., Plackett D. (2021). The Design, Characterization and Antibacterial Activity of Heat and Silver Crosslinked Poly(Vinyl Alcohol) Hydrogel Forming Dressings Containing Silver Nanoparticles. Nanomaterials.

[30] Lam E., Male K.B., Chong J.H., Leung A.C.W., Luong J.H.T. (2012). Applications of functionalized and nanoparticle-modified nanocrystalline cellulose. Trends Biotechnol..

[31] Thomas P., Duolikun T., Rumjit N.P., Moosavi S., Lai C.W., Bin Johan M.R., Fen L.B. (2020). Comprehensive review on nanocellulose: Recent developments, challenges and future prospects. J. Mech. Behav. Biomed. Mater..

[32] Peres B.U., Vidotti H.A., de Carvalho L.D., Manso A.P., Ko F., Carvalho R.M. (2019). Nanocrystalline cellulose as a reinforcing agent for electrospun polyacrylonitrile (PAN) nanofibers. J. Oral Biosci..

[33] Kummala R., Xu W., Xu C., Toivakka M. (2018). Stiffness and swelling characteristics of nanocellulose films in cell culture media. Cellulose.

[34] Liang L., Bhagia S., Li M., Huang C., Ragauskas A.J. (2020). Cross-Linked Nanocellulosic Materials and Their Applications. ChemSusChem.

[35] Curvello R., Raghuwanshi V.S., Garnier G. (2019). Engineering nanocellulose hydrogels for biomedical applications. Adv. Colloid Interface Sci..

[36] Hossain L., Raghuwanshi V.S., Tanner J., Wu C.-M., Kleinerman O., Cohen Y., Garnier G. (2020). Structure and swelling of cross-linked nanocellulose foams. J. Colloid Interface Sci..

[37] Jadhav K., Dhamecha D., Bhattacharya D., Patil M. (2016). Green and ecofriendly synthesis of silver nanoparticles: Characterization, biocompatibility studies and gel formulation for treatment of infections in burns. J. Photochem. Photobiol. B Biol..

[38] Morones-Ramirez J.R., Winkler J.A., Spina C.S., Collins J.J. (2013). Silver Enhances Antibiotic Activity Against Gram-Negative Bacteria. Sci. Transl. Med..

[39] Zhang X., Sun H., Tan S., Gao J., Fu Y., Liu Z. (2019). Hydrothermal synthesis of Ag nanoparticles on the nanocellulose and their antibacterial study. Inorg. Chem. Commun..

[40] Shin J.U., Gwon J., Lee S.Y., Yoo H.S. (2018). Silver-Incorporated Nanocellulose Fibers for Antibacterial Hydrogels. ACS Omega.

